# Effect of Biosynthesized ZnO Nanoparticles on Multi-Drug Resistant Pseudomonas Aeruginosa

**DOI:** 10.3390/antibiotics9050260

**Published:** 2020-05-17

**Authors:** Syed Ghazanfar Ali, Mohammad Azam Ansari, Mohammad A. Alzohairy, Mohammad N. Alomary, Mohammad Jalal, Sami AlYahya, Sarah Mousa Maadi Asiri, Haris M. Khan

**Affiliations:** 1 Department of Microbiology, Nanotechnology and Antimicrobial Drug Resistance Research Laboratory, Jawaharlal Nehru Medical College and Hospital, Aligarh Muslim University, Aligarh 202002, Uttar Pradesh, India; jalalmicro1981@gmail.com (M.J.); harismk2003@hotmail.com (H.M.K.); 2 Department of Epidemic Disease Research, Institute for Research & Medical Consultations (IRMC), Imam Abdulrahman Bin Faisal University, P.O. Box 1982, Dammam 31441, Saudi Arabia; 3 Department of Medical Laboratories, College of Applied Medical Sciences, Qassim University, Qassim 51431, Saudi Arabia; dr.alzohairy@gmail.com; 4 National Center for Biotechnology, Life Science and Environmental Research Institute, King Abdulaziz City for Science and Technology, P.O. Box 6086, Riyadh 11442, Saudi Arabia; malomary@kacst.edu.sa; 5 National Center for Biotechnology, King Abdulaziz City for Science and Technology, P.O. Box 6086, Riyadh 11442, Saudi Arabia; salyahya@kacst.edu.sa; 6 Department of Biophysics, Institutes for Research and Medical Consultations (IRMC), Imam Abdulrahman Bin Faisal University, Dammam 31441, Saudi Arabia; smasiri@iau.edu.sa

**Keywords:** *P. aeruginosa*, multidrug-resistant, quorum sensing, virulence factor, pyocyanin, ZnO NPs

## Abstract

Synthesis of nanoparticles using the plants has several advantages over other methods due to the environmentally friendly nature of plants. Besides being environmentally friendly, the synthesis of nanoparticles using plants or parts of the plants is also cost effective. The present study focuses on the biosynthesis of zinc oxide nanoparticles (ZnO NPs) using the seed extract of *Butea monsoperma* and their effect on to the quorum-mediated virulence factors of multidrug-resistant clinical isolates of *Pseudomonas aeruginosa* at sub minimum inhibitory concentration (MIC). The synthesized ZnO NPs were characterized by different techniques, such as Fourier transform infra-red spectroscopy (FTIR), scanning electron microscopy (SEM), energy dispersive X-ray (EDX), and transmission electron microscopy (TEM). The average size of the nanoparticles was 25 nm as analyzed by TEM. ZnO NPs at sub MIC decreased the production of virulence factors such as pyocyanin, protease and hemolysin for *P. aeruginosa* (*p* ≤ 0.05). The interaction of NPs with the *P. aeruginosa* cells on increasing concentration of NPs at sub MIC levels showed greater accumulation of nanoparticles inside the cells as analyzed by TEM.

## 1. Introduction

*Pseudomonas aeruginosa* is an opportunistic pathogen which attacks individuals suffering from different diseases including cancer, AIDS, and cystic fibrosis, as well patients who have medical implants or burn victims [1,2,3,4]. It is a very common pathogen that develops resistance against antibiotics and overcomes antibiotic treatment [5,6]. *P. aeruginosa* confers its pathogenesis and develops multidrug resistance through Quorum sensing [7]. Quorum sensing is a cell to cell communication responsible for different virulence gene expressions such as pyocyanin, proteases, toxins, and biofilm. Different compounds that interfere with this bacterial cell to cell communication are known as quorum quenchers, and these quorum quenchers attenuate the expressions of virulence genes responsible for proteases, toxins, siderophores, swarming and biofilm formation [8]. Quorum quenchers do not interfere with growth, and hence there is the least probability of development of resistance against them [9,10].

Nanotechnology is an emerging field since it has applications in science and technology for manufacturing new materials at the nanoscale level [11]. Nanotechnology at present is not only confined to the electronics, rather these nanoparticles posses a role in catalysis, magnetism, optical, and antimicrobial aspects [12,13], beside their application in wound healing and anti-inflammatory effects [14]. Metal oxide-like zinc oxide nanoparticles during the last few years have drawn great attention due to their stability and ability to overcome harsh environmental conditions. They can be easily fabricated even at low temperature via reflux digestion process [15] and are considered to be safe for human beings and animals [16,17]. Zn compounds have been currently listed as GRAS, generally regarded as safe by the US Food and Drug Administration (21CFRI82.8991).

There are different methods for the synthesis of nanoparticles including physical and chemical methods. Chemical and physical methods besides being costly require extensive labor, time, and also generate a large quantity of secondary waste from the chemical agents used for the precipitation and reduction. The green method of synthesis of nanoparticles has advantages over other methods including being cost efficient and eco-friendly. Since the green method of synthesis uses plant material as a capping agent so no adverse effect would be seen during medical applications [18]. Synthesis of different metal nanoparticles using different parts of plant extracts are well documented in the literature such as leaves of *Azadirachta indica* [19], leaves of *Putranjiva roxburghii* [20], stem of *Tinospora cordifolia* [21], coir of *Coccus nucifera* [22], Bark of *Crataeva nurvala* [23], bark extract of *Holarrhena pubescens* [24].

*Butea monosperma* (Lam.) Kuntze (BM) is commonly known as Flame of forest, because of its red colored flowers and belongs to the family Fabaceae. Single seed at the end locus is found inside the Pod and the length of each pod is around 10–15 cm. Due to their diverse nature, *B. monosperma* seeds were selected for the synthesis of ZnO NPs. Since the seeds of *B. monosperm*a are found inside the pods which fall down and contribute toward the waste material, therefore, we included seeds in this study. Furthermore, these biosynthesized zinc oxide nanoparticles were characterized by different sophisticated techniques, and further their effect on to the quorum sensing regulated virulence factors in *P. aeruginosa* was investigated.

## 2. Materials and Methods

### 2.1. Preparation of Aqueous Seed Extract of B. monosperma

The seeds of *B. monosperma* were obtained from the pods and in each pod a single seed was present. Seeds were collected from different pods and sun dried to remove the water content. The air dried seeds were crushed and ground to the fine powder. 10 g of powder was dissolved in 100 mL of sterile water, and then the aqueous extract was filtered using Whatman No. 1 filter paper (Maidstone, UK) and finally passed from 0.22 µm filter (Millipore). The centrifugation at 1200 rpm for 5 min removed the heavy biomaterials and extract in aqueous form was stored at 4 °C for further use.

### 2.2. Synthesis of B. monosperma Zinc Oxide Nanoparticles (BM-ZnO-NPs)

**Figure 1:**
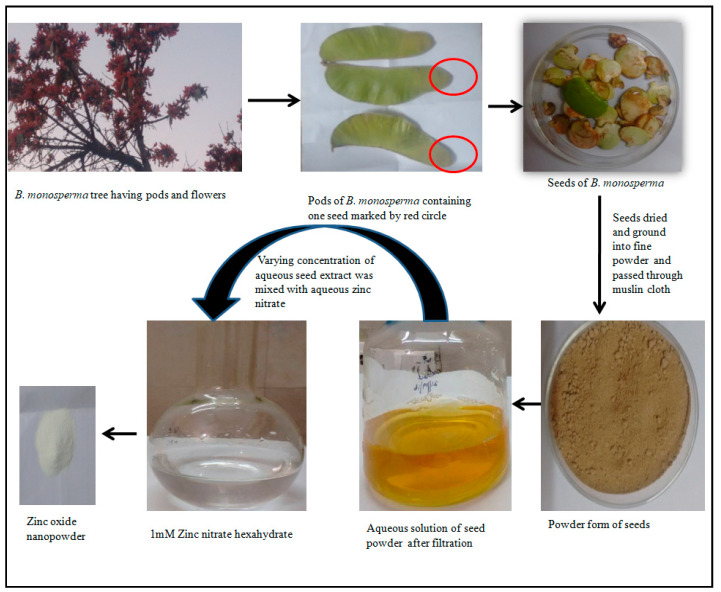
Diagrammatic representation of overall process of formation of ZnO NPs using *Butea monosperma* seeds.

Different volume (10 mL to 40 mL) of aqueous extract of *B. monosperma* seeds were mixed with 1mM Zinc nitrate hexahydrate solution. The reaction mixture was heated (60 °C) for 3–4 h until cream colored precipitate was not obtained. This cream color precipitate was further centrifuged at 3000 rpm for 15 min and the resulting pellet now obtained was collected in glass plate which was oven dried at 45 °C for 24–48 h. After complete drying, the powder obtained was further ground using mortar and pestle. The Diagrammatic representation of overall process of formation of ZnO NPs using *B. monosperma* seeds is represented in Figure 1.

### 2.3. Characterization of Synthesized BM-ZnO-NPs by FT-IR

FTIR spectrum was recorded using Perkin Elmer spectrophotometer spectrum in the range of 4000–400 cm^−1^ at room temperature (25–35 °C). The adsorption spectrum displayed different peaks representative of various bonds formed.

### 2.4. Scanning Electron Microscopy (SEM) and Energy Dispersive X-ray (EDAX)

To determine the morphology, distribution of nanoparticles and elemental composition of synthesized NPs, SEM equipped with EDAX(INCAx-actSN:56756) was performed. A thin film of nanoparticles was formed onto the glass coverslips by spreading the nanoparticles on to it, the samples were gold coated using gold coating sputter, and then the films were analyzed at an accelerating voltage of 15 KV.

### 2.5. Transmission Electron Microscopy (TEM)

The size and shape of the nanoparticles was further determined using TEM (Jeol 2100). Briefly, a copper grid was used onto which the sample (drop of nanoparticles) was placed, which was illuminated using electronic radiation under vacuum. Furthermore, the images were captured using electron beam transmitted through the sample [25].

### 2.6. Bacterial Isolates

*P. aeruginosa* (*N* = 10) were isolated from the patients’ samples (urine, pus, etc.) received in the department of Microbiology J.N. Medical College and Hospital. The *P. aeruginosa* were identified by using various biochemical tests and individual isolates were tested for the antibiotic sensitivity based on the recommendation of clinical and laboratory standards institute [26]. PAO1 was used as a standard.

### 2.7. Determination of Minimum Inhibitory Concentration (MIC) of ZnO NPs

Minimum inhibitory concentration of synthesized ZnO NPs was determined against *P. aeruginosa* by using two-fold macro broth dilution method as previously described [25]. Initially, *P. aeruginosa* colonies from overnight grown nutrient agar plates were used to inoculate the broth. Fresh overnight grown culture of *P. aeruginosa* (2 × 10^6^ CFU/mL) was used to inoculate each tube which was two-fold serially diluted with ZnO NPs at different concentration.

### 2.8. Inhibition of Quorum-Mediated Virulence Factors by BM-ZnO NPs

#### 2.8.1. Pyocyanin Assay

The effects of synthesized ZnO NPs on the production of virulence factor pyocyanin by *P. aeruginosa* were investigated using the methods described by Ali et al. [23] and Essar et al. [27]. Freshly grown overnight culture of *P. aeruginosa* was used to inoculate nutrient broth (5 mL) with or without varying concentration of BM-ZnO NPs in shaking incubator at 200 rpm. After incubation for 16 h at 37 °C, pyocyanin from the cell-free supernatant of the BM-ZnO-NPs treated and untreated *P. aeruginosa* culture was extracted with 3 mL chloroform and then re-extracted into 1 mL 0.2 N HCl and the absorbance was measure at 520 nm. The concentration of pyocyanin (µg) produced per ml of culture supernatant was determined by multiplying the optical density 520 nm by 17.072.

#### 2.8.2. Protease Assay

The protease assay was performed as described by Quiblier et al. [28] with slight modifications. Briefly, skim milk (5 gm) and bacteriological agar (0.5 gm) in 50 mL distilled water was amended with varying concentration of nanoparticles in different plates. Control plates were not amended with nanoparticles. An overnight grown culture of *P. aeruginosa* was put in the well of each plate and incubated overnight. Zone of clearance was measured [29]. Nutrient Broth was also added in each plate to check the proteolysis efficacy due to culture media.

#### 2.8.3. Hemolysis Assay

Hemolytic analysis was performed as described by Saghalli et al. [30] with slight modification. Initially, blood agar plates were prepared with 5% blood and 1% agar amended with the varying concentration of BM-ZnO-NPs and control plates were not amended with nanoparticles. Wells in the plates were punched, and gaps were sealed by soft agar. The overnight grown culture of *P. aeruginosa* including PAO1 was poured in wells in equal quantity in both the plates. Nutrient broth was also poured in both the plates to check the hemolysis due to media.

### 2.9. TEM Analysis Showing Localization of BM-ZnO NPs Inside Bacterial Cell

The effects and internalization of BM-ZnO NPs on *P. aeruginosa* cells before and after treatment has been analyzed by using TEM. The preparation of sample for TEM analysis such as fixation, dehydration, drying, coating, and imaging was performed as protocol described by Ansari et al. [25].

## 3. Results

### 3.1. FTIR Analysis

**Figure 2:**
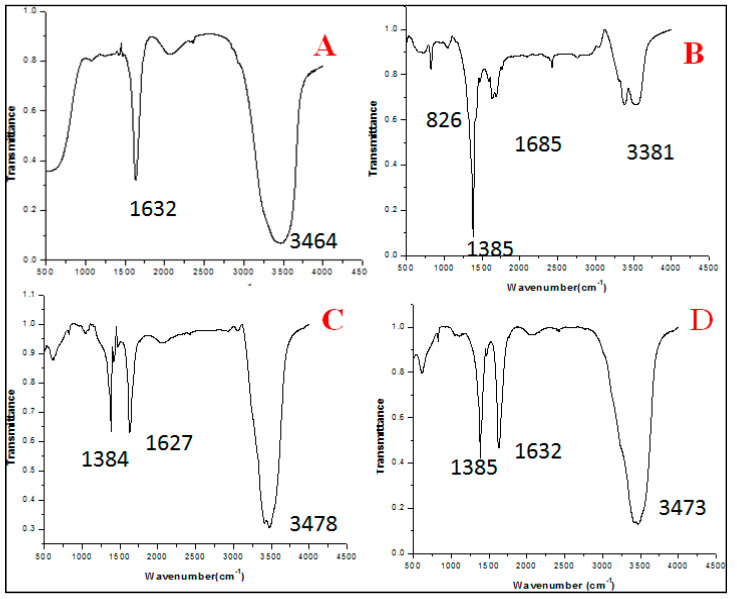
FTIR analysis between 4500–500 cm^−1^ showing different peaks which are representative of different bonds formed during the synthesis of zinc nanoparticles at different concentration of extract. (**A**) 10 mL extract, (**B**) 20 mL extract, (**C**) 30 mL extract, and (**D**) 40 mL extract.

The FTIR spectra at 10 mL extract concentration showed two different peaks at 1632, 3464 which represent -C=O stretching, -OH stretching whereas 20 mL extract concentration showed 4 intense peaks at 826, 1385, 1685, and 3381 correspondent to -C-H out of plane, -N-O(nitro), -NH_2_ wagging,-and NH stretch, respectively. Extract concentration at 30 mL showed three distinct peaks at 1384, 1627, 3478, representing N-O (nitro), -C=O (stretch), -O-H (stretch). 40 mL extract concentration also showed 3 peaks at 1385, 1632, 3473 for -N-O (nitro), -C=O, O-H (stretch), respectively (Figure 2).

### 3.2. SEM and EDAX Analysis

**Figure 3:**
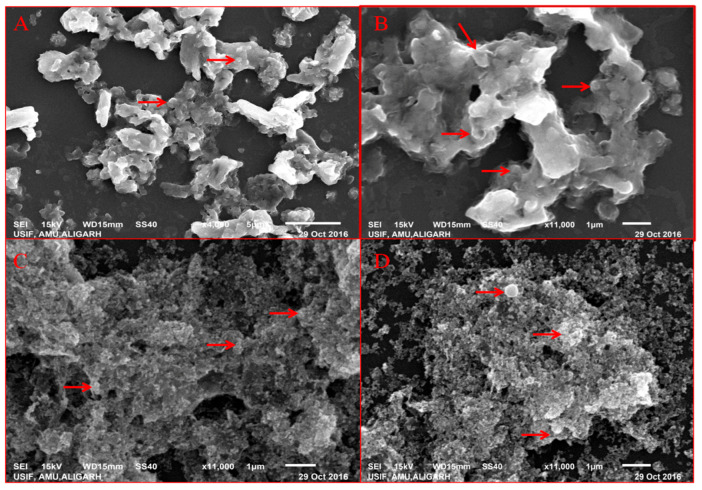
SEM of BM-ZnO NPs at different concentration of extract. (**A**) 10 mL, (**B**) 20 mL, (**C**) 30 mL, (**D**) 40 mL of extract.

**Figure 4:**
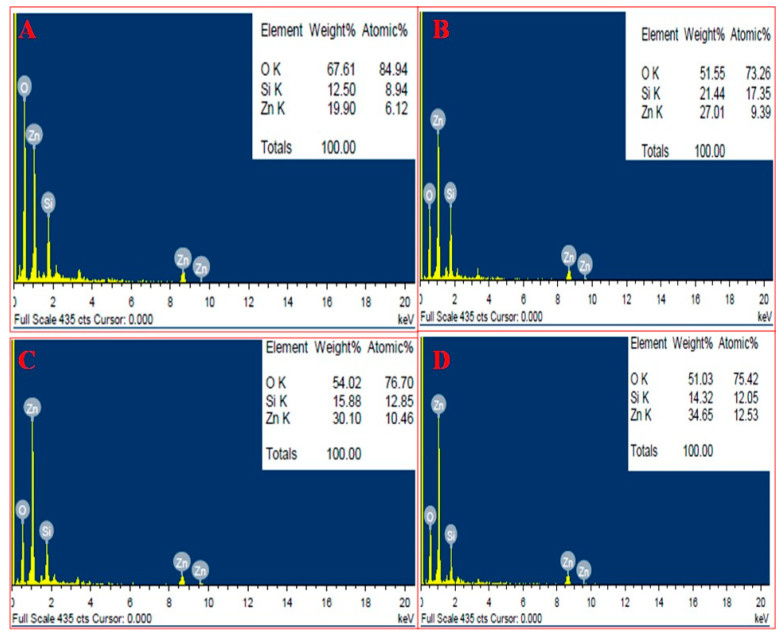
EDAX of BM-ZnO NPs at different concentration of extract. (**A**) 10 mL extract, (**B**) 20 mL extract, (**C**) 30 mL extract, and (**D**) 40 mL extract.

SEM analysis of nanoparticles at different concentration of extract showed the distribution of nanoparticles and their elemental composition was quantified by EDAX. SEM showed that nanoparticles at lower extract concentration (10 mL) were more clumped whereas on increasing the extract concentration segregation was seen, and at the highest concentration (40 mL) greatest segregation of nanoparticles was seen and individual nanoparticles were more clear (Figure 3). EDAX analysis showed that as the extract concentration increases the greater amount of ZnO NPs were formed, it is evident from the graphical analysis that at 10 mL of extract concentration 6.12% of zinc was analyzed whereas at 40 mL extract the number increased to 12.53% (Figure 4).

### 3.3. TEM Analysis of Synthesized ZnO NPs

**Figure 5:**
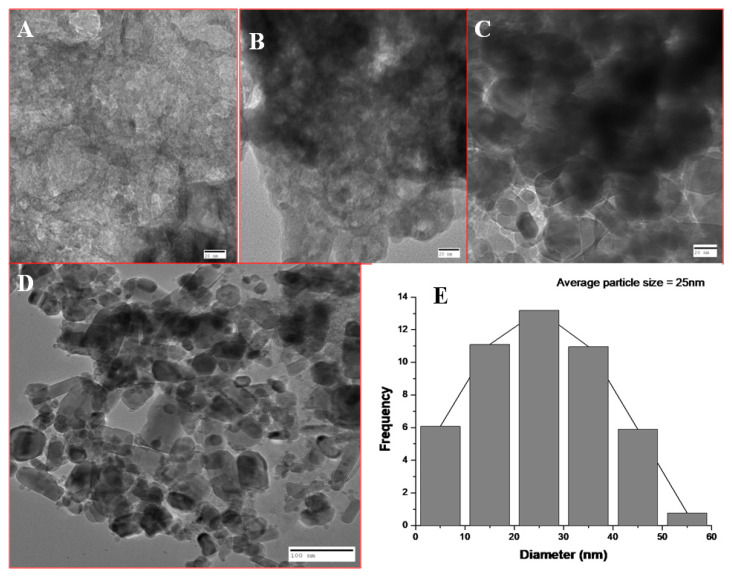
TEM of BM-ZnO NPs at different concentration of extract. (**A**) 10 mL extract, (**B**) 20 mL extract, (**C**) 30 mL extract, (**D**) 40 mL extract, (**E**) Average size of the nanoparticles at 40 mL extracts.

The average size of nanoparticles at 40 mL extract concentration was 25 nm as calculated by ImageJ software (Figure 5). The particle size at 10, 20, and 30 mL extract concentrations were not measured as there were greater aggregation and clumping of nanoparticles.

### 3.4. Antibiotic Resistance Pattern of Clinical Isolates of P. aeruginosa

All the 10 isolates of *P. aeruginosa* were resistant to Amikacin (Ak, 30 μg), cefepime (Cpm, 30 μg), sparfloxacin (Spx, 5 μg),piperacillin (Pi, 100 μg), Levofloxacin (Le, 5 μg), piperacilin-tazobactum (Pit, 100/10 μg), imipenem (Ipm, 10 μg), tobramycin (Tob, 10 μg), nitrofurantoin (Nit, 300 μg), and ceftazidime (Caz, 30 μg) antibiotics.

### 3.5. Minimum Inhibitory Concentration (MIC)

MIC value of BM-ZnO NPs against standard PAO1 was found to be 1600 µg/mL, whereas the MIC value ranged from 1600–3200 µg/mL for clinical isolates obtained from different sources (Table 1). Therefore, 300, 200, and 100 µg/mL were considered as sub MIC. 

### 3.6. Inhibition of Production of Quorum-Mediated Virulence Factors

**Figure 6:**
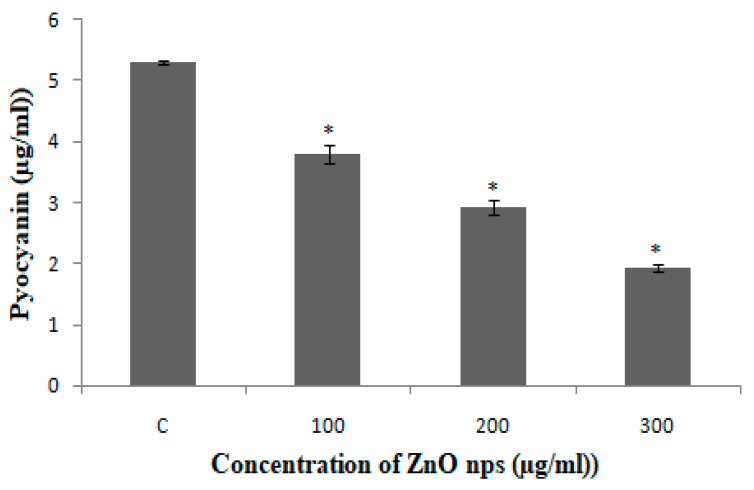
PAO1 treated with different concentration BM-ZnO NPs. Errors bars are indicative of standard deviation (±); * represents significance when *p* ≤ 0.05 & C represent control group.

**Figure 7:**
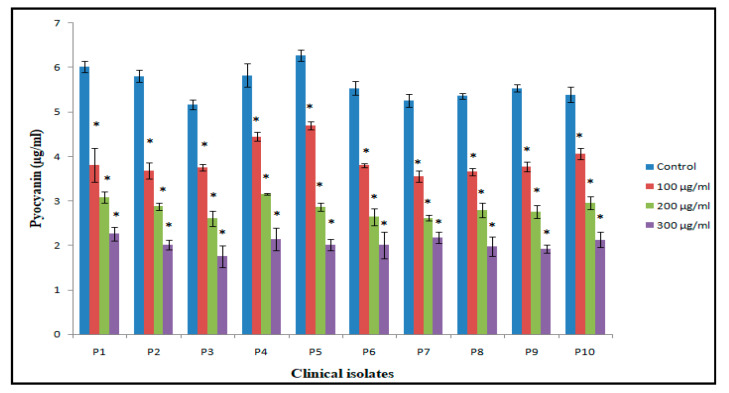
Bar graphs of clinical isolates of *P. aeruginosa* representing pyocyanin production (µg/mL) at three different concentrations (100, 200, 300 µg/mL) of BM-ZnO NPs along with control as untreated. * represents significance when *p* ≤ 0.05/mL; Error bars represent standard deviation.

**Figure 8:**
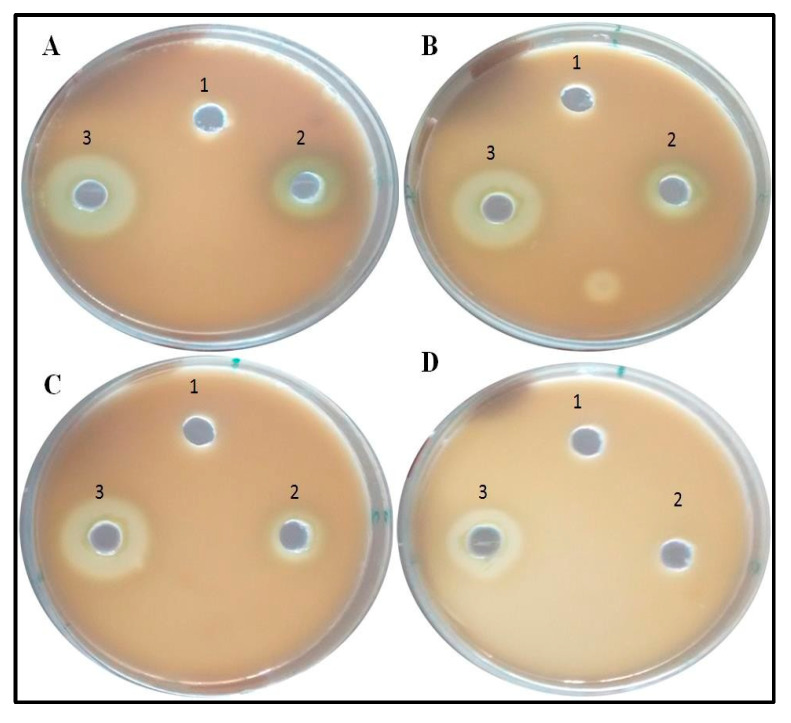
Images representative of Anti-proteolytic effect of ZnO NPs. Zone size decreases as the concentration increases. Well (1) represents Nutrient Broth, well (2) PAO1 culture, and well (3) Clinical isolate. (**A**) Control, (**B**) 100 µg/mL of ZnO amended, (**C**) 200 µg/mL of ZnO amended, (**D**) 300 µg/mL of ZnO amended.

**Figure 9:**
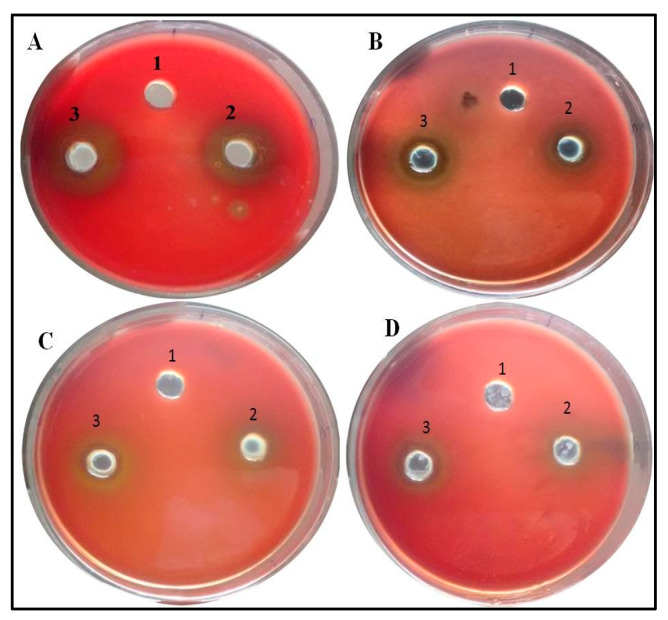
Images representative of Anti-hemolysis effect of ZnO NPs. Zone size decreases as the concentration increases. Well (1) represents Nutrient Broth, well (2) PAO1 culture and well (3) Clinical isolate. (**A**) Control, (**B**) 100 µg/mL of ZnO amended, (**C**) 200 µg/mL of ZnO amended, (**D**) 300 µg/mL of ZnO amended.

In case of PAO1, pyocyanin level decreased by 63.3% at 300 µg/mL, whereas 44.7% and 28.3% reduction was observed at 200 µg/mL and 100 µg/mL, respectively (Figure 6). The reduction in the level of pyocyanin for clinical isolates after treatment with 300 µg/mL of BM-ZnO NPs was 58.5% to 67.7%. The lower doses of ZnO NPs viz. 200 and 100 µg/mL also reduced the level of pyocyanin by 45.08% to 54.3% and 23.6% to 36.7%, respectively (Figure 7). Protease concentration for PAO1 reduced by 35.4% at 300 µg/mL, whereas 21.5% reduction was observed at 200 µg/mL. The sub MIC level of 100 µg/mL showed statistical insignificant results. In case of clinical isolates protease concentration decreased by 24.1% to 39.3% at300 µg/mL and at lower concentration of ZnO NPs viz. 200 µg/mL 11.2% to 30.3% reductions were observed (Figure 8). 100 µg/mL of concentration showed statistical insignificant results for all the isolates. Hemolytic activity of the isolates was also affected by ZnO NPs; PAO1 showed 45% reduction in the hemolytic activity at highest sub MIC level (300 µg/mL). However, at lower concentration i.e., 200 and 100 µg/mL, ZnO NPs reduced the hemolytic activity by 29.5% and 19.1%, respectively (Figure 9); whereas for clinical isolates 300 µg/mL of ZnO NPs successfully reduced the level of hemolytic activity in the range of 29.6% to 52.9% followed by 15.3% to 38.2% for 200 µg/mL and 14.1% to 29.4% at 100 µg/mL, respectively (Supplementary Materials Tables S1–S4).

### 3.7. TEM Analysis Showing Localization of BM-ZnO-NPs at Sub MIC Concentration

**Figure 10:**
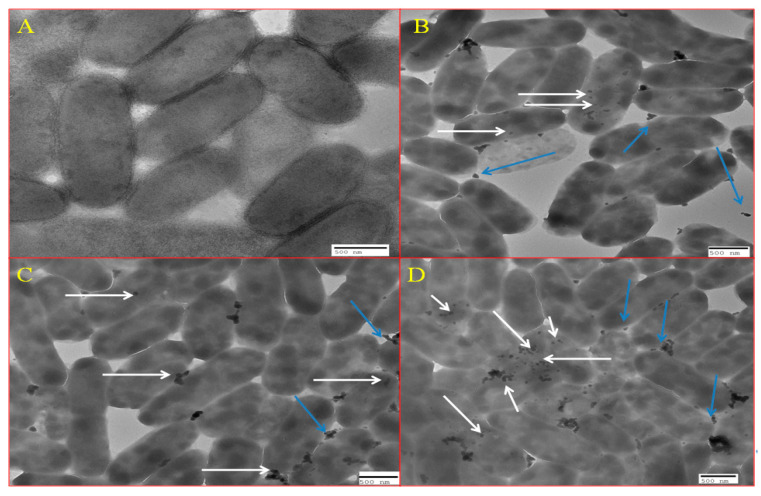
TEM showing internalization of ZnO NPs. (**A**) Control, (**B**) at 100 µg/mL of ZnO, (**C**) at 200 µg/mL of ZnO, and (**D**) 300 µg/mL of ZnO NPs. White arrows marks the internalized nanoparticles whereas blue arrow marks the nanoparticles which are outside the cells.

Figure 10A–D represents the localization of BM-ZnO-NPs inside the bacterial cells at sub MIC concentration i.e., 100, 200 and 300 µg/mL, respectively. It was observed that as the concentration of nanoparticles increased, there was a greater accumulation of NPs inside the cells.

## 4. Discussion

*P. aeruginosa* is a notorious pathogen causing nosocomial infections in individuals whose immune system has been weaken more specifically in burn patients [31,32]. It rarely causes community acquired infection in immuno-competent patients that is the reason it is known as an opportunistic pathogen. It is also evident from the literature that *P. aeruginosa* produces virulence factors and maintains multi drug resistance through quorum sensing [7]. Therefore the induction of quorum sensing inhibitors could be the key aspect in the anti-pathogenicity of this organism [33].

The present study demonstrates that ZnO NPs possess quorum-mediated anti-virulence property against the standard strain PAO1 as well as against multi drug resistant clinical isolates of *P. aeruginosa.*

### 4.1. Effect of Varying Volume of Extract on the Synthesis of Nanoparticles

Biosynthesized BM-ZnO NPs was characterized by different techniques viz. FTIR, SEM, EDAX, and TEM. FTIR analysis showed the formation of different bonds between extract and ZnO NPs. These bonds are remarkable features of the bio-molecules involved in the capping and stabilization of nanoparticles. SEM and TEM analysis showed distribution of particles and its aggregation, it is evident that higher proportion vs. nanoparticles posses more capping and stabilizing agent due to which greater uniformity in terms of shape and size was obtained, the fact is supported by the SEM and TEM analysis of ZnO NPs where greater uniformity was seen as the concentration of extract increased (Figure 3 and Figure 5). In other words, a greater amount of extract reduced precursor molecules (Zinc nitrate), which was confirmed by the EDAX analysis, which showed that a greater amount of zinc was obtained at higher extract concentration (Figure 4). Our results are also in agreement with previous studies which showed that on increasing extract concentration greater homogeneity regarding shape and size was obtained for zinc nanoparticles [34] and silver nanoparticles [35].

### 4.2. Effect of Bio Synthesized ZnO NPs on QS Mediated Virulence Factors

Pyocyanin, a blue, green phenazine pigment specifically produced by the *P. aeruginosa* [27], is directly operated by QS [1,36]. Pyocyanin, besides being the hallmark of *P. aeruginosa*, is also a secondary metabolite that leads to the severe toxic effect in combination with its precursor phenazine-1-carboxylic acid [37]. The damage caused by these two molecules accounts for the apoptosis of neutrophils and neutrophil mediated defensive mechanism [38]. BM-ZnO NPs decreased the production of pyocyanin in dose dependent manner as we observed that at the highest dose of 300 µg/mL of ZnO NPs there was the greatest decrease in production of pyocyanin as compared to the untreated cells (*p* ≤ 0.05). *P. aeruginosa* in order to maintain its pathogenicity and to further spread its infection secretes the hydrolytic enzymes such as hemolysin and proteases [39]. Proteases or peptidases are enzymes that degrade proteins and peptides by hydrolyzing the peptide bonds [40]. Proteins being the building block of cells when degraded by the protease affect the individual cells and further enhance the infection. Hemolysins leads to the lysis of host cell, different hemolysin such as Phospholipase and lecithinase acts in combination to break down lipids and lecithin and these proteins further encourage invasions by inducing cytotoxic effects on host cells [41]. BM-ZnO-NPs as shown in the result section decreased the production of hydrolytic enzymes viz. proteases and hemolysin. Maximum affect was seen at the highest dose (300 µg/mL) for PAO1 as well as for clinical isolates. Previous reports focused on to the antimicrobial aspect supporting the bacteriostatic or bactericidal approach [42] but very few reports focused on the antivirulence aspect supporting quorum quenching. Our results are in agreement with the previous work done by Garcia-Lara et al. [43] who showed that ZnO NPs affected the pyocyanin and elastase in *P. aeruginosa* at 100 µg/mL. They also proposed that virulence factors production and biofilm was due to quorum quenching effect of nanoparticles and not due to bacteriostatic or bactericidal effect. In another study, Saleh et al. [44] also reported that ZnO NPs can be effectively used as a quorum sensing inhibitor against *P. aeruginosa* infection and can be used as an alternative to conventional antimicrobials. Naseer Al Shabib [45] showed the similar results where *Nigella sativa* mediated ZnO NPs effectively inhibited the quorum regulated virulence factors in *P. aeruginosa* at sub MIC level.

Further, we are also of the opinion that due to the localization of BM-ZnO NPs inside the bacterial some mechanism would have been disturbed because of which virulence efficacy lowered down. Furthermore, the TEM analysis Figure 10A–D revealed that as the concentration of nanoparticles increased there was a greater accumulation of nanoparticles inside the cell and greater decrease in the virulence efficacy. 

## 5. Conclusions

The biosynthesized ZnO NPs at sub MIC level successfully inhibited the production of quorum sensing mediated virulence factors in *P. aeruginosa*. We are of opinion that ZnO NPs have quorum quenching effect due to which the virulence efficacy of *P. aeruginosa* lowered. Zinc oxide nanoparticles might have affected the QS mediated strategy either by arresting the production of QS molecules or by interacting with the QS molecules making them unidentified for the cell machinery and neutralizing their effect. Furthermore, studies on molecular aspect need to be done to know the exact mechanism involved in anti-virulence strategy, the studies on gene level could be a better option for knowing interaction between nanoparticles and gene expression.

## Figures and Tables

**Table 1:** MIC values of BM-ZnO NPs against *P. aeruginosa* (PAO1 and clinical isolates).

Isolates	MIC (μg mL^−1^)	Source
PAO1	1600	Standard
P1	3200	Pus
P2	3200	Pus
P3	1600	Pus
P4	3200	Pus
P5	1600	Pus
P6	3200	Urine
P7	3200	Pus
P8	3200	Urine
P9	1600	Pus
P10	3200	Urine

## Supplementary Materials

The following are available online at https://www.mdpi.com/2079-6382/9/5/260/s1, Table S1: Effect of different concentration of BM-ZnO NPs on different virulence factors of PAO1., Table S2: Effect of different concentration of BM-ZnO NPs on pyocyanin production by clinical isolates of *P. aeruginosa.*, Table S3: Effect of different concentration of BM-ZnO NPs on protease production by clinical isolates of *P. aeruginosa.*, Table S4: Effect of different concentration BM-ZnO NPs on hemolysis production by clinical isolates of *P. aeruginosa*.

## References

[1] De-Kievit T.R., Iglewski B.H. (2000). Bacterial quorum sensing in pathogenic relationships. Infect. Immun..

[2] Banu O., Bleotu C., Chifiriuc M.C., Savu B., Stanciu G.A., Antal C., Alexandrescu M., Lazar V. (2011). Virulence factors of *Staphylococcus aureus*and *Pseudomonas aeruginosa*strains involved in the etiology of cardiovascular infections. Biointerface Res. Appl. Chem..

[3] Saviuc C., Grumezescu A.M., Holban A., Bleotu C., Chifiriuc C., Paul B., Lazar V. (2011). Phenotypical studies of raw and nanosystem embedded *Eugenia carryophyllata*buds essential oil effect on *Pseudomonas aeruginosa*and *Staphylococcus aureus*strains. Biointerface Res. Appl. Chem..

[4] Andronescu E., Grumezescu A.M., Ficai A., Gheorghe I., Chifiriuc M., Mihaiescu D.E., Lazar V. (2012). In vitro efficacy of antibiotic magnetic dextran microspheres complexes against *Staphylococcus aureus*and *Pseudomonas aeruginosa*strains. Biointerface Res. Appl. Chem..

[5] Su H.C., Ramkissoon K., Doolittle J., Clark M., Khatun J., Secrest A., Wolfgang M.C., Gidding M.C. (2010). The development of ciprofloxacin resistance in *Pseudomonas aeruginosa*involves multiple response stages and multiple proteins. Antimicrob. Agents Chemother..

[6] Juan C., Zamorano L., Perez J.L., Ge Y., Oliver A. (2010). And on behalf of the Spanish Group for the Study of *Pseudomonas* and the Spanish Network for Research in Infectious Diseases Activity of a new anti pseudomonal cephalosporin, CXA-101 (FR264205), against carbapenem resistant and multi drug resistant *Pseudomonas aeruginosa*clinical strains. Antimicrob. Agents Chemother..

[7] Van-Delden C., Iglewski B.H. (1998). Cell-to-cell signaling and *Pseudomonas aeruginosa*infections. Emerg. Infect. Dis..

[8] Antunes L.C.M., Ferreira R.B.R., Buckner M.M.C., Finlay B.B. (2010). Quorum sensing in bacterial virulence. Microbiology.

[9] Defoirdt T., Boon N., Bossier P. (2010). Can bacteria evolve resistance to quorum sensing disruption?. PLoS Pathog..

[10] Rasko D.A., Sperandio V. (2010). Anti-virulence strategies to combat bacteria-mediated disease. Nat. Rev. Drug Discov..

[11] Albrecht M.A., Evans C.W., Raston C.L. (2006). Green chemistry and the health implications of nanoparticles. Green Chem..

[12] Duran N., Marcato P.D., Alves O.L., De Souza G.I.H., Esposito E. (2005). Mechanistic aspects of biosynthesis of silver nano particles by several Fusariumoxysporum strains. J. Nanobiotecnol..

[13] Ingle A., Gade A., Pierrat S., Sonnichsen C., Mahendra R. (2008). Mycosynthesis of silver nanoparticles using the fungus *Fusariumacuminatum*and its activity against some human pathogenic bacteria. Curr. Nanosci..

[14] Taylor P.L., Ussher A.L., Burrell R.E. (2005). Impact of heat on nanocrystalline silver dressings. Part I: Chemical and biological properties. Biomaterials.

[15] Dhage S.R., Pasricha R., Ravi V. (2005). Synthesis of fine particles of ZnO at 100 °C. Mater. Lett..

[16] Stoimenov P.K., Klinger R.L., Marchin G.L., Klabunde K.J. (2002). Metal oxide nanoparticles as bactericidal agent. Langmuir.

[17] Fu L., Liu Z., Liu Y., Han B., Hu P., Cao L., Zhu D. (2005). Beaded Cobalt oxide nanoparticle along carbon nanotubes: Towards more highly integrated electronic devices. Adv. Mater..

[18] Kharissova O.V., Dias H.V.R., Kharisov B.I., Perez B.O., Jimenez Perez V.M. (2013). The Greener Synthesis of Nanoparticles. Trends Biotechnol..

[19] Shankar S.S., Rai A., Ahmad A., Sastry M. (2004). Rapid synthesis of Au, Ag, and bimetallic Au core–Ag shell nanoparticles using Neem (*Azadirachta indica*) leaf broth. J. Colloid Interface Sci..

[20] Ali S.G., Khan H.M., Jalal M., Ansari M.A., Mahdi A.A., Ahmad M.K. (2015). Green synthesis of silver nanoparticles using the leaf extract of *Putranjiva roxburghii* wall. and their antimicrobial activity. Asian J. Pharm. Clin. Res..

[21] Ali S.G., Ansari M.A., Alzohairy M.A., Alomary M.N., AlYahya S., Jalal M., Khan H.M., Asiri S.M.M., Ahmad W., Mahdi A.A. (2020). Biogenic Gold Nanoparticles as Potent Antibacterial and Antibiofilm Nano-Antibiotics against *Pseudomonas aeruginosa*. Antibiotics.

[22] Roopan S.M., Rohita, Madhumitha G., Rahuman A.A., Kamaraj C., Bharathi A., Suredra T.V. (2012). Low-cost and eco-friendly phyto-synthesis of silver nanoparticles using *Cocosnucifera*coir extract and its larvicidal activity. Ind. Crops Prod..

[23] Ali S.G., Ansari M.A., Khan H.M., Jalal M., Mahdi A.A., Cameotra S.S. (2017). Crataeva nurvala nanoparticles inhibit virulence factors and biofilm formation in clinical isolates of Pseudomonas aeruginosa. J. Basic Microbiol..

[24] Ali S.G., Ansari M.A., Khan H.M., Jalal M., Mahdi A.A., Cameotra S.S. (2018). Antibacterial and antibiofilm potential of green synthesized silver nanoparticles against imipenem resistant clinical isolates of P. aeruginosa. Bio. Nano Sci..

[25] Baig U., Gondal M.A., Ansari M.A., Akhtar S. (2018). Facile synthesis, characterization and antibacterial activity of nanostructured palladium loaded silicon carbide. Ceram. Int..

[26] Clinical Laboratory Standards Institute (2012). Performance Standards for Antimicrobial Susceptibility Testing.

[27] Essar D.W., Eberly L., Hadero A., Crawford I.P. (1990). Identification and characterization of genes for a second anthranilate synthase in *Pseudomonas aeruginosa*: Interchangeability of the two anthranilate synthases and evolutionary implications. J. Bacteriol..

[28] Quiblier C., Zinkernagel A.S., Schuepbach R.A., Berger-Bächi B. (1990). Contribution of SecDF to Staphylococcus aureus resistance and expression of virulence factors. BMC Microbiol..

[29] Jalal M., Ansari M.A., Alzohairy M.A., Ali S.G., Khan H.M., Almatroudi A., Siddiqui M.I. (2019). Anticandidal activity of biosynthesized silver nanoparticles: Effect on growth, cell morphology, and key virulence attributes of Candida species. Int. J. Nanomed..

[30] Saghalli M., Bidoki S.K., Jamali A., Bagheri H., Ghaemi E.A. (2017). Sub-minimum inhibitory concentrations of Zinc Oxide Nanoparticles Reduce the Expression of the Staphylococcus aureus Alpha-Hemolysin. Indian J. Pharm. Sci..

[31] Costerton J.W., Stewart P.S., Greenberg E.P. (1999). Bacterial biofilms: A common cause of persistent infections. Science.

[32] Whiteley M., Bangera M.G., Bumgarner R.E., Parskek M.R., Teitzel G.M., Lory S., Greenberg E.P. (2001). Gene expression in Pseudomonas aeruginosa biofilms. Nature.

[33] Fothergill J.L., Winstanley C., James C.E. (2012). Novel therapeutic strategies to counter *Pseudomonas aeruginosa*infections. Expert Rev. Anti Infect. Ther..

[34] Nava O.J., Luque P.A., Gomez-Gutierrez C.M., Vilchis-Nestor A.R., Castro-Beltran A., Mota- Gonzalez M.L., Olivas A. (2017). Influence of *Camellia sinensis*extract on Zinc Oxide nanoparticlegreen synthesis. Mol. Struct..

[35] Khalil M.M.H., Ismail E.H., El-Baghdady K.Z., Mohamed D. (2014). Green synthesis of silver nanoparticles using olive leaf extract and its antibacterial activity. Arab. J. Chem..

[36] Karatuna O., Yagci A. (2010). Analysis of quorum sensing dependent virulence factor production and its relationship with antimicrobial susceptibility in *Pseudomonas aeruginosa*respiratory isolates. Clin. Microbiol. Infect..

[37] Fothergill J.L., Panagea S., Hart C.A., Walshaw M.J., Pitt T.L., Winstanlet C. (2007). Widespread pyocyanin over production among isolates of a cystic fibrosis epidemic strain. BMC Microbiol..

[38] Usher L.R., Lawson R.A., Geary I., Taylor C.J., Bingle C.D., Taylor G.W., Whyte M.K. (2002). Induction of neutrophil apoptosis by the *Pseudomonas aeruginosa*exotoxin pyocyanin: A potential mechanism of persistent infection. J. Immunol..

[39] Gupta R.K., Setia S., Harjai K. (2011). Expression of quorum sensing and virulence factors is interlinked in P. aeruginosa: An in vitro approach. Am. J. Biomed. Sci..

[40] Barrett A.J., Woessner J.F., Rawlings N.D. (2004). Handbook of Proteolytic Enzymes.

[41] Wilson R., Pitt T., Taylor G., Watson D., Mac Dermot J., Sykes D., Roberts D., Cole P. (1987). Pyocyanin and 1-hydroxyphenazine produced by *Pseudomonas aeruginosa*inhibit the beating of human respiratory cilia in vitro. J. Clin. Investig..

[42] Sirelkhatim A., Mahmud S., Seeni A., Kaus N.H., Ann L.C., Bakhori S.K., Hasan H., Mohamad D. (2015). Review on zinc oxide nanoparticles: Antibacterial activity and toxicity mechanism. Nano-Micro Lett..

[43] Garcia-Lara B., Saucedo-Mora M.A., Roldan-Sanchez J.A., Perez-Eretza B., Ramasamy M., Lee J., Coria-Jimenez R., Tapia M., Varela-Guerrero V., Garcia-Contreras R. (2015). Inhibition of quorum-sensing-dependent virulence factorsand biofilm formation of clinical and environmental Pseudomonas aeruginosa strains by ZnO nanoparticles. Lett. Appl. Microbiol..

[44] Saleh M.M., Sadeq R.A., Abdel Latif H.K., Abbas H.A., Askoura M. (2019). Zinc oxide nanoparticles inhibits quorum sensing and virulence in Pseudomonas aeruginosa. Afr. Health Sci..

[45] Al-Shabib N.A., Husain F.M., Ahmed F., Khan R.A., Ahmad I., Alsharaeh E., Khan M.S., Hussain A., Rehman M.T., Yusuf M. (2016). Biogenic synthesis of Zinc oxide nanostructures from Nigella sativa seed: Prospective role as food packaging material inhibiting broad-spectrum quorum sensing and biofilm. Sci. Rep..

