# Supplementary material for: Effect of Biosynthesized ZnO Nanoparticles on Multi-Drug Resistant Pseudomonas Aeruginosa
Source: Antibiotics (Basel). 2020 May 17;9(5):260. doi: 10.3390/antibiotics9050260 (PMC7277366; doi:10.3390/antibiotics9050260)
Supplement: Supplementary file 1 [file antibiotics-09-00260-s001.pdf]

Supplementary Materials

Table S1**:** Effect of different concentration of BM-ZnO NPs on different virulence factors of PAO1.

PAO1 (Treated with ZnO NPs)				
		Treatment (μg/mL)		
**Virulence factors**	**Control**	**100 (μg/mL)**	**200 (μg/mL)**	**300 (μg/mL)**
Pyocyanin	5.30 ± 0.04	3.80 ± 0.15 *	2.93 ± 0.13 *	1.94 ± 0.06 *
Protease	18.6 ± 1.15	17.3 ± 2.51	14.6 ± 0.57 *	12 ± 1 *
Hemolysin	19.3 ± 1.1	15.6 ± 1.1 *	13.6 ± 0.57 *	10.6 ± 1.15 *

Pyocyanin concentration is represented as μg/mL; Protease and Hemolysin is represented as zone of lysis in mm, ± means standard deviation, * means significant values $p \leq 0.05$.

Table S2: Effect of different concentration of BM-ZnO NPs on pyocyanin production by clinical isolates of *P. aeruginosa*.

Pyocyanin				
**Isolates**	**Control**	**Treatment (μg/mL)**		
		100	200	300
P1	6.02 ± 0.13	3.81 ± 0.38 *	3.09 ±0.13 *	2.26 ± 0.16 *
P2	5.81 ± 0.14	3.68 ± 0.18 *	2.88 ±0.08 *	2.01 ± 0.11 *
P3	5.16 ± 0.11	3.75 ± 0.07 *	2.61 ±0.17 *	1.75 ± 0.25 *
P4	5.83 ± 0.26	4.45 ± 0.10 *	3.16 ±0.23 *	2.14 ± 0.25 *
P5	6.27 ±0.13	4.69 ± 0.09 *	2.86 ±0.09 *	2.02 ± 0.13 *
P6	5.54 ± 0.16	3.8 ± 0.05 *	2.64 ±0.19 *	2.01 ± 0.30 *
P7	5.26 ± 0.15	3.55 ±0.13 *	2.62 ±0.06 *	2.18 ± 0.13 *
P8	5.36 ± 0.06	3.66 ±0.08 *	2.8 ± 0.16 *	1.98 ± 0.21 *
P9	5.54 ± 0.08	3.78 ±0.11 *	2.76 ± 0.14 *	1.93 ± 0.09 *
P10	5.39 ± 0.17	4.06 ± 0.13 *	2.96 ± 0.11 *	2.13 ± 0.17 *

Pyocyanin concentration is expressed as μg/mL; ± standard deviation; * means significant values $p \leq 0.05$.

Table S3: Effect of different concentration of BM-ZnO NPs on protease production by clinical isolates of *P. aeruginosa*.

Protease				
**Isolates**	**Control**	**Treatment (μg/mL)**		
		**100**	**200**	**300**
P1	22 ± 2	18.66 ± 0.57	15.33 ± 1.15 *	13.33 ± 1.15 *
P2	24 ± 2	21.66 ± 1.52	18.33 ± 1.52 *	15.33 ± 0.57 *
P3	19.33 ± 1.15	17.33 ± 1.15	15.33 ± 1.15 *	13 ± 1 *
P4	21 ± 1	18.33 ± 0.57	16 ± 1 *	14.33 ± 1.15 *
P5	21.66 ± 1.52	20± 2	18 ± 2 *	15 ± 1.73 *
P6	21.66 ± 1.52	20.33± 0.57	18.33 ± 1.52 *	15 ± 1 *
P7	20.66 ± 1.52	19± 1	16.66 ± 1.15 *	14 ± 1 *
P8	20.33 ± 0.57	18.66± 2.08	17 ± 2 *	13.66 ± 1.52 *
P9	21.33 ± 1.15	19.33± 0.57	18 ± 2 *	15 ± 1 *
P10	20.66 ± 1.52	19 ± 1.57	18.33 ± 1.52 *	15.66 ± .57 *

Protease concentration mentioned as zone of lysis in mm; ± means standard deviation, * means significant values $p \leq 0.05$.

Table S4: Effect of different concentration BM-ZnO NPs on hemolysis production by clinical isolates of *P. aeruginosa*.

Hemolysin				
**Isolates**	**Control**	**Treatment (μg/mL)**		
		**100**	**200**	**300**
P1	22.66 ± 1.15	16 ± 1 *	14 ± 1*	10.66 ± 1.15 *
P2	25.33 ± 1.15	18 ±2 *	16 ± 1*	12.33 ± 0.57 *
P3	19.66 ± 1.52	16.8 ±1 *	15 ± 1*	13 ± 1 *
P4	20.66 ± 1.15	16.66 ± 1.15 *	14.66 ± 1.15 *	11.66 ± 1.52 *
P5	22 ± 2	20.33 ± 0.57 *	17.33 ± 1.15 *	14 ± 1 *
P6	21.66 ± 0.57	20.33 ± 0.57 *	18.33 ± 1.52 *	15 ± 1 *
P7	20.66 ± 1.15	19 ± 1 *	16.66 ± 1.15 *	14 ± 1 *
P8	20.33 ± 1.52	18.33 ± 1.52 *	17 ± 2 *	13.66 ± 1.52 *
P9	21.33 ± 1.15	19.33 ± 0.57 *	18 ± 2 *	15 ± 1 *
P10	21.66 ± 1.15	18.66 ± 0.57 *	16 ± 1 *	13.33 ± 0.57 *

Hemolysis activity mentioned as zone of lysis in mm; ± means standard deviation; * means significant values $p \leq 0.05$.
